# Genomic diversity and population structure analysis of four geographically distinct Holstein cattle populations using genome-wide DNA profiling

**DOI:** 10.5194/aab-68-565-2025

**Published:** 2025-09-11

**Authors:** Ronak Salehi, Arash Javanmard, Mahdi Mokhber, Sadegh Alijani

**Affiliations:** 1 Department of Animal Science, Faculty of Agriculture, University of Tabriz, Tabriz, Iran; 2 Department of Animal Science, Faculty of Agriculture, Urmia University, Urmia, Iran

## Abstract

Holstein cattle are the world's most widely distributed dairy breed, present in more than 150 countries. Despite their large population and widespread presence, the genomic diversity of Holstein subpopulations across the globe is surprisingly low; therefore, genetic monitoring of these populations, especially in countries that import their genetic material, is crucial. This study aimed to compare the genomic diversity of Iranian Holstein cattle with populations from France, Poland, and Sweden, providing a comprehensive overview of the Holstein breed's genetic structure. In total, 399 Holstein cattle from four geographically distinct countries, namely, Iran ( n=25 ), France ( n=153 ), Poland ( n=198 ), and Sweden ( n=23 ), were selected for analysis. Quality control and data filtering were conducted using PLINK 1.9. After quality control, a pruning process was applied to the genomic data, resulting in 10 822 single-nucleotide polymorphism markers for genomic diversity analysis. Due to limitations in the software used for population structure analysis, missing data were imputed using Beagle 5.2. To analyze the genetic structure of the populations, discriminant analysis of principal components (DAPC) and population admixture analysis were performed using the adegenet package in R. Moreover, genomic diversity and distances were assessed using Weir and Cockerham's unbiased differentiation index ( $F_{\mathrm{ST}}$ ) and Nei's genetic distance. The results were visualized using R software. The DAPC analysis revealed minimal genetic differences among the studied populations. Based on the findings, the studied populations from Iran, France, Sweden, and Poland formed a single genetic group with no significant differentiation. These results were further confirmed through $F_{\mathrm{ST}}$ and Nei's genetic distance statistic and population admixture analysis. Overall, a high genetic similarity was found between Iranian Holstein cattle and Holstein cattle populations in other countries, particularly in Europe and North America. This similarity is likely due to the extensive importation of genetic material and the absence of an effective breeding and selection program in Iran. Future studies may investigate the precise influence of these factors on the genomic diversity and population structure of the Holstein breed. The results of this study can be used to explore the structure of the Holstein population. Likewise, they can be beneficial in national breeding schemes and planning for the importation of genetic material (especially semen).

## 1 Introduction

Throughout the history of animal husbandry, cattle – as large ruminants – have been widely dispersed and successfully adapted to a broad range of climatic conditions. Since their domestication, cattle have become an essential source of food and labor for humans (Papachristou et al., 2020). In 2022, the global cattle population reached approximately 1.55 billion heads, up from around 1.51 billion heads in 2021 (FAO, 2023). The Holstein Friesian is the most widespread breed across more than 150 countries, forming a large metapopulation, and is currently the leading dairy breed worldwide (Vostry et al., 2023).

The Holstein Friesian is the most widespread breed across more than 150 countries, forming a large metapopulation, and is currently the leading dairy breed worldwide.

The genomic diversity of Holstein cattle is relatively low despite its large population and wide distribution. For instance, the effective population size (Ne) of Holstein populations in countries such as Australia, Canada, Denmark, Spain, Ireland, and the USA ranges between 49 and 127 (Doekes et al., 2018). The low genomic diversity in this breed can be attributed to several factors. Among the main reasons are the intense selection and overuse of a small number of top-performing bulls, particularly those with higher milk production potential (Yue et al., 2015). Additionally, the high gene flow and strong connections between subpopulations have further diminished genomic diversity within the Holstein metapopulation (Ablondi et al., 2022; Doekes et al., 2018). As a result, managing genomic diversity and inbreeding has become a key focus in many national Holstein breeding programs (Rodríguez-Ramilo et al., 2015).

Understanding the genomic diversity of domestic species is essential for the development of effective management strategies and genetic conservation (Wultsch et al., 2016). Recent advancements in genome sequencing and the availability of high-density genomic data have facilitated the analysis of genomic diversity and population structure (Decker et al., 2014). Methods such as genetic distances, population clustering, and admixture analysis are commonly used to assess genomic diversity within populations (Al-Mamun et al., 2015).

In recent years, a growing body of research has focused on the genomic diversity of various domesticated species, including cattle, buffalo, goats, and sheep. The findings of different studies (e.g., Moradi Shahrbabak et al., 2023; Rahimmadar et al., 2021; Colli et al., 2018; Doekes et al., 2018; Mastrangelo et al., 2018; Kukučková et al., 2017; Rodríguez-Ramilo et al., 2015; Gautier et al., 2010) have provided valuable insights into the genetic variation within these populations. Additionally, some studies have focused on the genomic diversity of goats and sheep (e.g., Giovannini et al., 2024; Kusza et al., 2024), contributing to further understanding of the genetic composition of these domesticated animals. Despite its global prominence, the Holstein breed faces challenges in maintaining genomic diversity due to the extensive use of semen from elite bulls and artificial insemination practices. Therefore, the present study seeks to compare the genomic diversity of Iranian Holstein cattle with populations from Poland, France, and Sweden, providing a thorough overview of the genetic structure of the Holstein breed.

## 2 Material and methods

### 2.1 Animals and source of raw data

A total of 399 Holstein cattle from four geographically distinct countries, including Iran ( n=25 ), France ( n=153 ), Poland ( n=198 ), and Sweden ( n=23 ), were meticulously selected based on the study purpose. Genomic information used in this study was obtained through reliable online databases, including Dryad and WIDDE (Sempéré et al., 2015), or through electronic correspondence with relevant researchers (Table 1).

**Table 1:** Genomic data of six used Holstein populations related to four countries: Iran, France, Poland, and Sweden.

No.	Mark	Country	Sample	Quick access address – genomic array	Relevant article
			no.		
1	IRI	Iran	25	Illumina_BovineSNP50v2	Shakeri et al. (2021)
2	FRA_LD	France	30	https://doi.org/10.1371/journal.pone.0013038 & WIDDE	Gautier et al. (2010)
3	FRA_LD	France	63	https://doi.org/10.1371/journal.pone.0005350 & WIDDE Illumina_BovineSNP50v1	Matukumalli et al. (2009)
					
4	FRA_HD	France	60	http://widde.toulouse.inra.fr/widde/widde/main.do;jsessionid=EB8D8557C0E6C34E3040E06D29FDA729?module=cattle (last access: January 2023) & WIDDE umina.com/applications/agriculture.ilmn Illumina BovineHD	Project: ILLUMINA_HD
5	POL	Poland	198	Illumina_BovineSNP50v1	Requested
6	SWE	Sweden	23	–	Salehi et al. (2023b)
Total			399		

### 2.2 Quality control and filtering process

Data quality control and filtering were performed using PLINK 1.9, following the protocols outlined by Purcell et al. (2007) and Toro-Ospina et al. (2022). Loci located on sex chromosomes, loci with unknown chromosomal locations, single-nucleotide polymorphism (SNP) loci with call rate genotyping (CR_SNP_) less than 95 % and minor allele frequency (MAF) less than 5 %, and SNP markers that were out of Hardy–Weinberg equilibrium ( P value less than 10^−6^) were excluded during this process. Similarly, individuals with more than 5 % missing genotypes were also excluded from the analysis. Subsequently, data aggregation and pruning were performed, resulting in 10 822 SNP markers for genomic diversity analysis. Due to the limitations of the software for population structure analysis, missing genotypes were imputed using Beagle software (Browning et al., 2021).

### 2.3 Genomic diversity statistical analysis

The genomic diversity and racial groups were studied using principal component analysis (PCA) and genetic group determination methods (Elhaik, 2022; Ma and Amos, 2012). To investigate the genetic structure of the population and its constituent subpopulations, discriminant analysis of principal components (DAPC) was conducted using the adegenet package in R software (Jombart and Ahmed, 2011). Moreover, Weir and Cockerham's differentiation index (1984) and genetic distances were calculated in the R environment (Toro-Ospina et al., 2022; Purcell et al., 2007). Furthermore, the adegenet package in the R environment was used to study the populations' genetic commonality and analyze population structure in greater detail (Jombart and Ahmed, 2011). Eventually, visual representations of the analysis results were created using the R environment (R Core Team, 2024).

## 3 Results

### 3.1 PCA and DAPC methods

**Figure 1:**
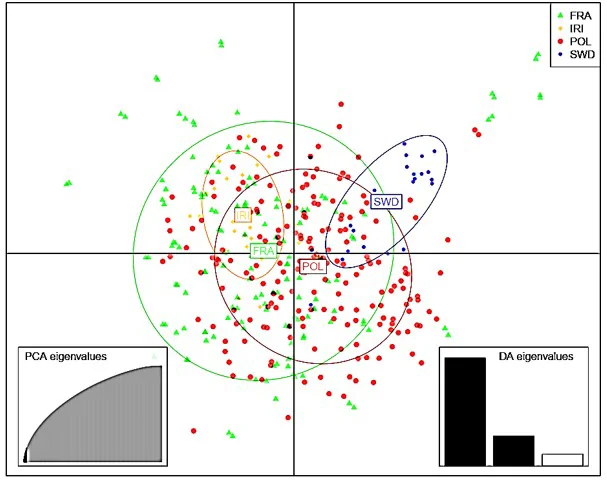
PCA results of discriminant analysis of principal components of Holstein cattle populations in Iran, France, Poland, and Sweden. PCA: principal component analysis; DA: discriminant analysis.

**Figure 2:**
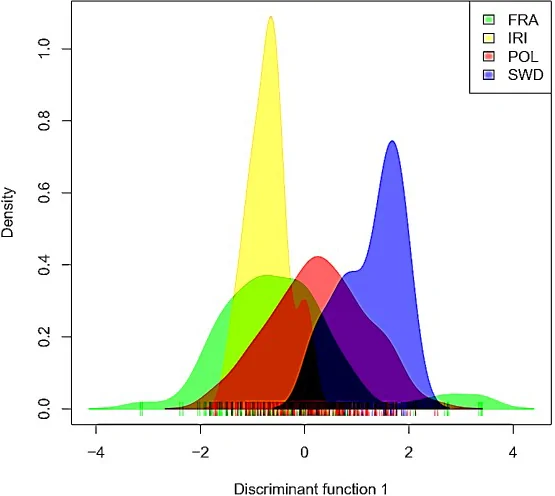
Advanced clustering outputs of discriminant analysis of principal components of Holstein cattle populations in Iran, France, Poland, and Sweden.

Initially, the data were subjected to PCA (Price et al., 2006), followed by discriminant analysis to identify clusters according to the method described in previous research (Jombart and Ahmed, 2011). To determine the proximity of populations and their genetic relationships, information on identity by descent and identity by state was obtained from the genomic relationship matrix (Villanueva et al., 2021; Makgahlela et al., 2013). Figures 1 and 2 display the graphical representation of the separation of the studied Holstein cattle populations using SNP markers. The results of the DAPC indicated a lack of distinct clusters and groups in both cases (Figs. 1 and 2). The study utilized information from components 1–40 to deduce genetic grouping. Interestingly, the findings revealed that increasing the number of components resulted in greater differentiation between individuals and groups. Nonetheless, slight differentiation was observed among the studied groups when using the information from the first 40 components to draw graphs related to genetic grouping. This observation is attributed to the diversity being primarily related to subpopulations within the Holstein breed.

In the analysis, the results emphasized the significance of the first component (Fig. 2), suggesting potential population mixing or a shared origin among the studied animals. The observed diversity within and between populations was found in both visual representations. Based on the results (Fig. 2), the French population exhibited the highest genomic diversity, whereas the Iranian population exhibited the lowest genomic diversity.

### 3.2 Genetic distance across populations

**Figure 3:**
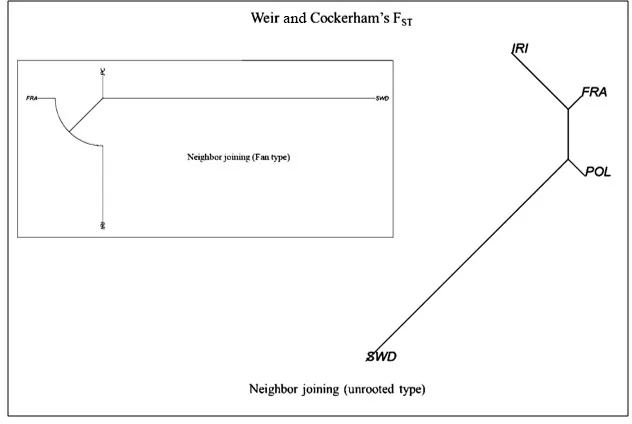
Phylogeny tree of Holstein cattle populations in Iran, France, Poland, and Sweden.

To further analyze population diversity, genetic distances were calculated, and a phylogenetic tree was constructed for the studied populations. The outcomes of these calculations are presented in Table 2 and Fig. 3. The findings revealed significant variations between the populations. Furthermore, the analysis of population differentiation using the Weir and Cockerham differentiation index and the examination of genetic distances conform to the findings of the DAPC analysis.

**Table 2:** Genetic distances of Holstein cattle populations in Iran, France, Poland, and Sweden.

Populations	FRA	IRI	POL	SWD
FRA	0.00	0.0038	0.0030	0.0144
IRI	0.0038	0.00	0.0066	0.0156
POL	0.0030	0.0066	0.00	0.0120
SWD	0.0144	0.0156	0.0120	0.00

### 3.3 Structure-based allele admixture analysis

**Figure 4:**
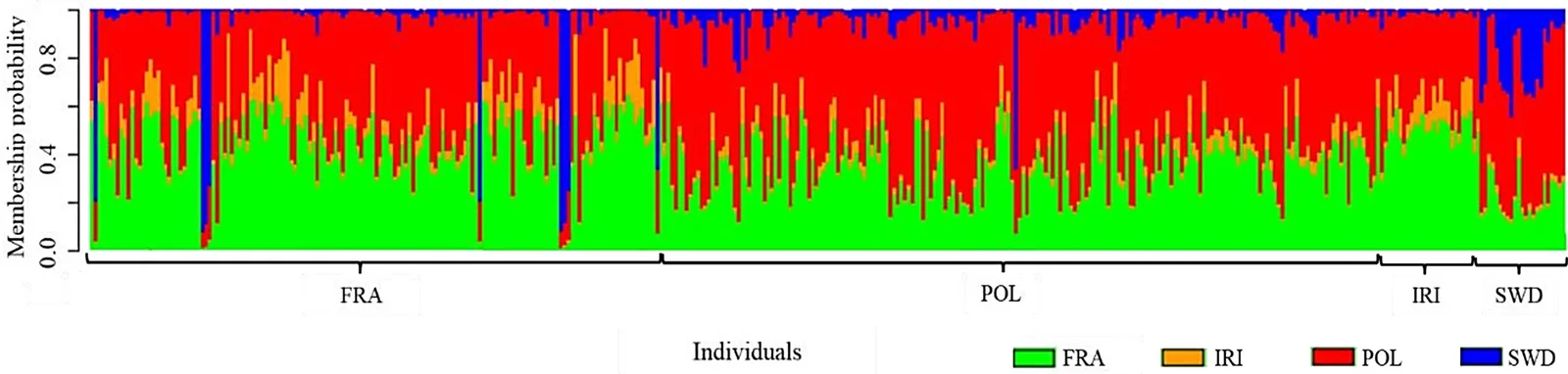
Graphical diagram of shared genomic diversity related to the overall appearance of the genome of each population of Holstein cattle with geographical differences.

The results (Fig. 4) confirmed the genetic mixing of the studied populations, serving as a supplementary analysis for genomic diversity studies. The findings in this section reaffirm the results of previous analyses. While this analysis is primarily utilized for studying the genetic mixing of different populations, it also demonstrates genomic similarities and can be applied to individuals within the same population. The genetic commonalities of each of the studied animals are separately presented, with a unique color assigned to each population to indicate the contribution of each individual's genome from the studied populations. In this analysis, a specific color, or “ K equals 4”, was designated for the number of studied populations. Assuming a K value lower than 4 would be possible to discuss similarities at the level of one or two genetic groups. However, in this instance, a value of 4 was chosen to discern the minimal differences through a more in-depth examination of the population.

The observed pattern indicates a significant genetic contribution from the French and Polish populations to Holstein cattle. Iranian Holsteins demonstrate a strong genetic affinity with the French population, followed by Polish Holsteins, with a close genetic affinity. Notably, the Swedish population exhibits the highest genetic divergence from the other three populations. It is worth mentioning that these genetic affinities reflect a shared ancestry, likely influenced by the historical movement of Holstein cattle from various countries. This genetic insight underscores the substantial impact of international exchanges in shaping the Holstein cow genome on a global scale.

## 4 Discussion

In this study, Weir and Cockerham's $F_{\mathrm{ST}}$ (1984) and Nei's genetic distance (1972) were selected as primary measures of genetic differentiation. These metrics are widely used for SNP-based diversity studies due to their robustness and ability to quantify population-level structure (Allendorf et al., 2013). While other metrics such as heterozygosity or allelic richness offer valuable insights, they can be more sensitive to sample size and may not fully reflect inter-population divergence (Kalinowski, 2004; Luikart et al., 2013). Our goal was to evaluate genetic differentiation across populations, for which $F_{\mathrm{ST}}$ and Nei's distance remain standard and well-validated tools. However, we acknowledge their limitations in capturing within-population diversity and recommend future studies to complement them with additional metrics as DAPC and population admixture analyses.

### 4.1 PCA and DAPC methods

Understanding the population's genetic structure is crucial for various purposes, such as identifying stratifications in livestock populations, conducting genome-wide association studies, designing breeding programs, and developing genetic resource conservation strategies. Our study used DAPC analysis, population differentiation index, genetic distance determination, and population mixing to assess the genetic structure. The DAPC method is a multivariate approach that helps identify and describe clusters of genetically related individuals. The reduced genomic diversity in the Holstein breed can be attributed to factors such as shared ancestry, rigorous selection practices, and a relatively small effective population size worldwide. The initial 10 components (Fig. 1) were utilized during the compilation of the genetic clustering analysis.

As illustrated in Fig. 1, the studied livestock populations exhibit a shared genetic origin with no clear signs of significant differentiation. The visual analysis of the generated graphs shows that intra-population diversity substantially contributes to overall diversity. Furthermore, the populations of France and Poland exhibit higher diversity than those of Iran and Sweden. The observed diversity can be attributed to the higher number and variety of samples from the respective populations.

The DAPC method has been widely employed in numerous studies (Kusza et al., 2024; Moradi Shahrebabak et al., 2023; Sharma et al., 2016) to assess genomic diversity. It is important to emphasize that the first two principal components provide a reliable measure of differentiation among populations and individuals within each population (Gaspar and Breen, 2019; Kijas et al., 2013; Jombart et al., 2010). However, leveraging additional component information enhances the accuracy and interpretability of the results. The decision to include the number of components in population differentiation analysis and genetic grouping is contingent upon the diversity of the populations under study.

Within the scope of the current study, the first two components accounted for 18 % of the variance, while the first 10 components collectively explained 25 % of the total variance. Despite justifying the same amount of variance, no significant population discrimination was recognizable. Conversely, in the investigation performed by Biabani et al. (2022) on genomic diversity between Holstein and native Iranian livestock, the studied populations were distinctly categorized, with the first two components elucidating 74.9 % of the variance. Moreover, Salehi et al. (2023a) observed justified variances of 12.87 and 5.44 for the first and second components, respectively.

In the study by Sharma et al. (2016), the justified variance of the initial 100 components equated to 52 % of the total variance.

### 4.2 Genetic distance across populations

Based on the genetic distance analysis results, the population of France demonstrated the lowest distance compared to the populations of Poland (0.0030) and Iran (0.0038), while the genetic distance between Poland and Iran was 0.0066. Additionally, the Swedish population exhibited genetic distances ranging from 0.0120 to 0.0156 compared to other populations, placing the French population between the Iranian and Polish populations. Notably, previous studies reported a high level of genetic compatibility between two subsets of data pertaining to the French population, namely, a high-density series (60 heads) and a low-density series (93 heads), which were considered inseparable in genetic terms (Salehi et al., 2023b). In this analysis, both datasets were collectively evaluated as a single population. Salehi et al. (2023b) further highlighted that the utilization of high-density arrays (777 962 SNPs) is unnecessary for the assessment of genomic diversity, and genomic information obtained from lower-density arrays (the Illumina BovineSNP50 series, typically comprising ∼ 54 000 markers) was adequate.

Genetic distance, a measure of the genetic differentiation between populations, can be quantified using the genetic differentiation index ( $F_{\mathrm{ST}}$ ). These values are categorized based on numerical intervals (Wright, 1978) into low (0–0.05), medium (0.15–0.05), high (0.25–0.15), and very high (more than 0.25) groups. They provide a classification system for reporting the genetic relationships between populations. According to Wright (1978), low $F_{\mathrm{ST}}$ values signify a close genetic relationship between populations. In the context of the studied Holstein populations, low $F_{\mathrm{ST}}$ values suggest minimal genetic differentiation, indicating a common origin and high gene flow between the populations.

In a comprehensive study on the genomic diversity of the Holstein population, Vostry et al. (2023) examined 2178 Holstein heads from 12 European and American Holstein subpopulations, along with the Simmental breed as an outgroup. Their phylogenetic analysis revealed interesting findings. They found that the Holstein populations of Belgium, England, Canada, the Netherlands, France, Germany, the Czech Republic, and the United States were grouped together, while the subpopulations of Switzerland and Ireland, as well as Croatia, were distinct from this main group. The genetic differentiation of the Simmental breed from the Holstein subpopulations was found to be in the numerical range of 0.049–0.060, while differentiation within the Holstein subpopulations showed two distinct intervals. The difference between Croatia, Ireland, and Switzerland populations compared to other Holsteins (Belgium, England, Canada, the Netherlands, France, Germany, Czech Republic, and the USA) was in the numerical range of 0.011–0.028. Moreover, the distance among the Holstein populations of Belgium, England, Canada, the Netherlands, France, Germany, Czech Republic, and the USA was in the numerical range of 0.001–0.008. The results and the research objectives of the present study are consistent with those of Vostry et al. (2023).

The above study compared the Holsteins of the Czech Republic with the Holstein populations of other countries and reported that the bulls used in artificial insemination in the Czech Republic differed from those in other countries, especially regarding genes affecting meat and carcasses. In addition, despite the common origin of the world's Holsteins, each country had adopted different selective paths, depending on its breeding goals, leading to relative differentiation of Holstein populations. This differentiation was particularly evident in the Swedish population, which was highly different from other studied populations, including Iran, France, and Poland. Additionally, the high similarity of the Iranian Holstein population with other genetic groups suggests high kinship and gene flow with the Iranian population (Vostry et al., 2023).

### 4.3 Structure-based allele admixture analysis

The observed pattern indicated a significant genetic contribution from the French and Polish populations to Holstein cattle. Iranian Holsteins demonstrated a strong genetic affinity with the French population, while Polish Holsteins represented a close genetic affinity. Notably, the Swedish population exhibited the greatest genetic divergence from the other three populations. Notably, these genetic affinities reflect a shared ancestry, likely influenced by the historical movement of Holstein cattle from various countries. This genetic insight underscores the substantial impact of international exchanges in shaping the Holstein cow genome on a global scale. In a separate investigation exploring the genomic diversity of Holsteins worldwide, data from China, Canada, the Netherlands, and Ireland were also included (Salehi et al., 2023b). However, due to the varying data formats for these populations, it was impossible to analyze the genetic differences alongside the data from the current study. The high differentiation of genetic groups could be attributed to the incompatible data formats with those of the present study. The division of cattle breeds globally relies on exploring the genetic differentiation between cattle breeds with *Bos taurus* and *Bos indicus* backgrounds (Ben Jemaa et al., 2015; Deckers et al., 2014), indicating that the origin or genetic background serves as a fundamental factor in the differentiation of cattle populations worldwide. Additionally, geographical distribution is another crucial factor in the racial differentiation of livestock populations. This is attributed to genetic differentiation among groups being influenced by their geographical distribution and the varying climatic conditions of their breeding regions (Berihulay et al., 2019).

The results of the present study regarding the genomic data of French Holstein were compared with those of the study by Vostry et al. (2023). This comparison could provide a better understanding of the genomic diversity of Iranian Holstein cows in relation to other European and American Holstein populations. To thoroughly discuss the genomic diversity of the Holstein population in Iran, it is important to investigate the origins of the genetic material. The low genetic distance between the French Holstein population and that of Iran in this study (0.0038) indicates a high likelihood of strong genetic relationships between Iranian Holsteins and other Holstein populations worldwide, including the USA, Ireland, Germany, the Netherlands, and Canada. This suggests that Iranian Holsteins maintain a very close genetic relationship with commercial Holstein populations worldwide. The genetic purity of Iranian Holsteins appears to be high, closely resembling the genetic profile of Holsteins from large commercial farms. Genetic resources for Iranian Holsteins are likely derived from external sources, particularly in cases where the genetic material is obtained from international sperm sources. Moreover, the low level of genetic differentiation in Iranian Holsteins indicates that targeted breeding efforts have not substantially shaped their genomic structure. Geographic isolation and racial mixing may have contributed to the observed genetic differences between populations (Vostry et al., 2023; Berihulay et al., 2019).

The genomic diversity of Holstein cattle worldwide has decreased due to extensive breeding programs and selection, reducing the effective population size. Studies reported that the effective population size values for Holstein cattle in several countries ranged from 49 to 127 heads. This decrease in genomic diversity is not surprising, given the extensive exchange of genetic material and the high relatedness of this breed globally. Despite the high genetic connections, differences can be observed in the national diversity of Holstein cows due to selection programs and genetic drift in different regions of the world. For example, the genomic diversity of Danish Holstein has increased in recent years due to breeding programs. Additionally, some studies compared the genetic distances between Holstein subpopulations and other dairy and beef breeds worldwide, including indigenous cows of different regions. Karimi et al. (2016) found that the genetic differentiation of Iranian native cattle with Holstein varies from 0.129 in Kurdi to 0.332 in Sistani. Interestingly, the genetic differentiation of some native Iranian breeds with Holstein is less than the differentiation between native Iranian breeds. For example, Moradi Shahrebabak et al. (2023) reported the genetic differentiation of Sistani and Kurdish cows as 0.18 and Sistani and Sarabi as 0.166. Based on Moradi Shahrebabak et al. (2023) findings, the differences observed in the study groups of Holstein populations and native breeds of Iran were related to genetic background and geographical differentiation. Sistani cattle belong to the *Bos indicus* subspecies and have different living conditions from the *Bos taurus* breed, such as the Kurdish breed (Moradi Shahrebabak et al., 2023). Likewise, Makina et al. (2014) compared the genetic distance between Holstein and Angus with several native breeds from South Africa. The genetic distance between Holstein and Angus was 0.098, while the distance between Holstein and the native breeds of South Africa was reported to be up to 0.159.

Recent advancements in population genetics have raised important concerns about the sustainability of current Holstein breeding strategies in Iran. The widespread use of elite sires through artificial insemination has led to significant improvements in productivity. However, this strategy has also resulted in increased inbreeding and a reduction in effective population size, which in turn has caused a decline in genetic diversity – a crucial factor for long-term adaptability (Doekes et al., 2018; Vostry et al., 2023). To address these challenges, future strategies should focus on broadening the genetic base by incorporating less-related or underutilized lineages, including locally adapted bulls. Establishing a national genomic selection program and regularly monitoring diversity indices – such as inbreeding coefficients and runs of homozygosity – can help guide informed selection while preventing excessive genetic relatedness (Doekes et al., 2018). Additionally, the cryopreservation of genetically diverse germplasm could serve as a valuable resource for future breeding flexibility and resilience (Vostry et al., 2023).

The findings from this study highlight the genetic homogenization of Iranian Holstein cattle due to the widespread use of imported semen from a narrow elite pool. While this approach has improved production traits, it has also raised concerns for long-term sustainability by reducing genetic diversity and effective population size. To mitigate these risks and preserve genetic resilience, Iran's breeding strategies should prioritize genomic monitoring, diversify sire origins, and incorporate local selection programs that are tailored to national needs (Ameri et al., 2023; Vostry et al., 2023).

## 5 Conclusions

Examining the genetic variation at the genomic level provides valuable insights into the differentiation between populations and individuals within a population. This information is crucial for breeders aiming to enhance livestock performance and for genetic conservation strategists. In this study, the Iranian Holstein cattle population's genomic diversity was comprehensively analyzed compared to that of the French, Polish, and Swedish Holstein populations. The findings revealed the genetic affinity of Iran's purebred Holstein cattle population with that of other countries that primarily export genetic material of this breed. The minimal difference between Iran's Holstein cattle and other global Holstein populations, particularly those in Europe and the United States, can be attributed to the substantial import of genetic material from these regions. Additionally, it reflects historical breeding practices and the lack of effective breeding programs in Iran. These results provide a meaningful foundation for understanding population structure and contribute to the refinement of breeding programs and the development of effective conservation plans. Furthermore, the findings can inform national decisions pertaining to the dairy cattle breeding industry, specifically in determining the sources of genetic material input (sperm) in the country. Future investigations are essential to confirm and expand upon the conclusions drawn from this study.

## Data Availability

The datasets used and/or analyzed during the current study are available from the corresponding author on reasonable request.
